# Assessment of low-coverage nanopore long read sequencing for SNP genotyping in doubled haploid canola (*Brassica napus* L.)

**DOI:** 10.1038/s41598-019-45131-0

**Published:** 2019-06-18

**Authors:** M. M. Malmberg, G. C. Spangenberg, H. D. Daetwyler, N. O. I. Cogan

**Affiliations:** 1 Agriculture Victoria, AgriBio, Centre for AgriBioscience, 5 Ring Road, Bundoora, Victoria 3083 Australia; 2 School of Applied Systems Biology, La Trobe University, Bundoora, Victoria 3086 Australia

**Keywords:** Plant genetics, DNA sequencing, Next-generation sequencing

## Abstract

Despite the high accuracy of short read sequencing (SRS), there are still issues with attaining accurate single nucleotide polymorphism (SNP) genotypes at low sequencing coverage and in highly duplicated genomes due to misalignment. Long read sequencing (LRS) systems, including the Oxford Nanopore Technologies (ONT) minION, have become popular options for *de novo* genome assembly and structural variant characterisation. The current high error rate often requires substantial post-sequencing correction and would appear to prevent the adoption of this system for SNP genotyping, but nanopore sequencing errors are largely random. Using low coverage ONT minION sequencing for genotyping of pre-validated SNP loci was examined in 9 canola doubled haploids. The minION genotypes were compared to the Illumina sequences to determine the extent and nature of genotype discrepancies between the two systems. The significant increase in read length improved alignment to the genome and the absence of classical SRS biases results in a more even representation of the genome. Sequencing errors are present, primarily in the form of heterozygous genotypes, which can be removed in completely homozygous backgrounds but requires more advanced bioinformatics in heterozygous genomes. Developments in this technology are promising for routine genotyping in the future.

## Introduction

*Brassica napus* is a recent allotetraploid arising from natural hybridisation events between *B. rapa* and *B. oleracea*, with high collinearity to both progenitor genomes^1^. As such, not only are the progenitor genomes highly homoeologous, but there is also extensive within genome duplication due to numerous ancestral duplication events including a *Brassiceae* specific triplication event^2,3^, and frequent homoeologous recombination^4^. This high level of duplication across the genome leads to misalignment of short sequencing reads, complicating the distinction between true single nucleotide polymorphisms (SNPs) and differentiation between sub-genomes or paralogues. In a comparison between two cultivars, c. 90% of SNPs identified were found to be caused by variation between homoeologous regions and were termed “hemi-SNPs”^5^. Short probe-based methods face similar issues, with up to 62% of SNPs in a pair of doubled haploids (DHs) found to be heterozygous when using the *Brassica* 6 K SNP array^6^. In fact, it is common practise when using the *Brassica* SNP arrays to remove SNP loci whose flanking sequence aligns to multiple regions in the reference genome, typically leaving 20–30 K SNPs from the 60 K array which are informative and can be uniquely aligned^7^. Numerous studies in canola, as well as other polyploid or paleopolyploid crop species including soybean, maize and peanut, have encountered this issue and routinely apply filtering strategies aimed at removing SNPs caused by misalignment in an attempt to minimise the number of false-positive SNPs^8–16^, demonstrating the difficulty of accurately genotyping highly duplicated genomes. Ultimately, the accuracy of association studies and genomic prediction is increased when all present variation is captured, including heterozygotes^17–19^.

Long read sequencing (LRS) technology generates reads which may span far enough to uniquely align to a reference genome. LRS has quickly become the technology of choice for *de novo* genome assembly^20–25^ and identification of structural variants due to the ability to span repetitive regions and complete variants^26–29^. However, the current error rate of c. 10%^22,30,31^, requires the use of more accurate short read sequencing (SRS) to polish such assemblies to ensure the final sequence is correct and has likely prevented the assessment of LRS for routine SNP genotyping purposes. Since skim whole genome re-sequencing (skim WGR) using SRS has been found to be highly cost-effective, high-throughput and a relatively accurate genotyping-by-sequencing method^32–35^, LRS must be sufficiently accurate at skim levels without correction in order to compete with current SRS technology. The SRS data generated for error correction would likely in itself be sufficient for genotyping purposes in many species. In addition, performing such error correction has the potential to introduce the same misalignment errors which are typical of SRS, as polishing involves the alignment of SRS to LRS. While skim WGR using LRS is probably currently unsuitable for *de novo* SNP discovery without correction or substantial sequencing depth, there is the potential that LRS can accurately genotype pre-determined SNP positions, as long as alignment algorithms are able to correctly break and align reads to a reference genome around short insertions and deletions, which are the predominant form of error in ONT minION sequencing^30^. However, for minION LRS to be realistically used for genotyping purposes, the resulting genotype calls need to contain fewer errors than the overall 10% error rate, although this may be tolerable for genomic selection, which has been found to be relatively robust to error rates of up to c. 10%^36^.

The steady decline in the cost of sequencing coupled with the advantages of a whole genome approach suggests that WGR will become the predominant form of genotyping in the future, whether based on LRS or SRS. At current cost structures and outputs, neither the PacBio or ONT LRS can compete with the Illumina HiSeq/NovaSeq systems. However, developments in these technologies are rapid and the ONT promethION system is projected to rival Illumina HiSeqxTEN system in the near future (https://nanoporetech.com/resource-centre/videos/sub1000). Although the PacBio system is absent of systemic errors and the circular consensus of fragments between 3 and 20 kb can be highly accurate, the ONT minION and promethION systems are preferable due to superior cost structures, sequencing outputs and ease of library preparation that typically takes less than one day. And while the nanopore base-calling algorithm does have some systemic errors, the majority of errors are non-systemic^31^ and can be improved with more sequencing depth, unlike in Illumina sequencing, and future improvements to quality scoring based on the behaviour of these systemic errors will further minimise this issue. Additionally, existing third-party software can be applied and does not rely on platform provided pipelines, also suggesting future independent development of genotyping pipelines targeted at nanopores sequencing.

The aim of this study was to perform an initial evaluation of nanopore LRS for skim WGR genotyping of pre-defined SNP positions, as compared to commonly used Illumina SRS, to determine whether minION LRS could reasonably be used instead of Illumina SRS based genotyping. Nine canola DHs were used due to the simpler genetic background and were compared to the genotypes of the same samples generated from Illumina. As any routine genotyping technique will require multiplexing to be cost-effective, the native barcoding kit followed by 1D ligation library preparation was used to generate sequences on the ONT minION sequencer. This study aimed to i) assess the effect of long reads on misalignment to the reference genome, ii) determine if the overall confidence in the resulting genotypes can be improved by applying various filtering treatments, and iii) describe the nature of discrepancies between Illumina and minION sequencing derived genotypes.

## Results

### MinION run QC

A total of four minION sequencing runs were prepared from the same extracted genetic material and were processed for library preparation twice. Runs 1 and 2 were generated from the same pool prepared using the 108 chemistry and runs 3 and 4 from the same pool using the 109 chemistry, with the final adapter ligation performed just prior to each sequencing run. A reduction in yield is observed in the second runs (runs 2 and 4) and a minor reduction in mean Q score in run 4 (Table S1), probably due to degradation of the library in the time between sequencing runs. Across all samples, a total of 3,561,686 reads passed Q score filtering and had an average length of c. 7,500 bp and an average Q score of 9.8 (Table S1).

### Alignment of long and short reads

**Figure 1:**
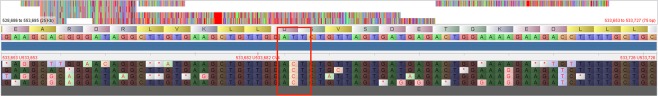
Tablet view of alignment of minION sequencing reads to the Darmor-*bzh* reference genome, with known SNP (chr A01 position 533686) in DH-9 outlined.

Without additional filtering, the percentage of sequencing reads which align to the reference genome is similar between Illumina and minION, but this figure reduces to between 51.1% and 66.0% when a minimum q score of 30 is applied to the Illumina sequences, while between 97.5% and 98.8% of minION reads, which are not filtered based on q score, aligned to the reference genome (Table S2). Visual inspection of aligned minION reads shows that sequencing errors are prevalent but mostly randomly spread throughout the read and that minION sequences can produce clear genotype signals at known SNP positions (Fig. 1). As described in the materials and methods, these known SNP positions were previously identified and validated in a large set of relatively deeply sequenced canola genomes, removing the need to perform *de novo* SNP discovery.

### Assessing the accuracy of minION genotype calls compared to Illumina

The validity of using minION sequencing for genotype calling is assessed by comparing the genotype calls at known SNP positions between Illumina SRS and minION LRS generated in the same 9 DH samples. Any genotype call which is concordant between the two systems is deemed to be accurate. Genotype calls which are discordant between the two systems are generally assumed to be true in the Illumina sequences, due to the greater accuracy of Illumina SRS. However, as these are DH samples which are expected to be fully homozygous, this study suggests that genotype calls which are homozygous minION but heterozygous in Illumina are more likely to be correct in the minION sequences. Unless otherwise specified, references to “accurate” genotype calls refers to calls which are consistent between the two sequencing systems.

### Effect of LRS on misalignment in DH canola

The 9 samples which were sequenced are DHs, such that no heterozygous genotypes are expected. To determine the effect of both sequencing systems on misalignment, the percentage of heterozygous genotypes in each individual was examined in the Illumina and minION sequencing, both filtered for a read depth between 2 and 5 for consistency. As well as an examination of all combined minION runs, each sequencing run was examined separately, especially as runs 3 and 4 were unevenly pooled, resulting in more variable genome coverage between individuals, which allows the association between sequencing coverage and heterozygosity to be examined. The average percentage of heterozygous genotype calls was similar between the Illumina data and all combined minION runs, at 4.6% and 4.2% respectively (Table 1). However, in the minION sequencing, there is highly significant negative correlation between coverage and heterozygosity in runs 3 and 4, which demonstrate variable coverage. This suggests that most heterozygotes in minION genotypes are caused by sequencing errors, as the addition of more sequencing data drowns out these errors as noise. All combined minION runs display variable genome coverage and moderate but non-significant correlation with heterozygosity. Runs 1 and 2 have no significant correlation with coverage, perhaps caused by high consistency in coverage preventing a clear correlation signature from being detected. In contrast, in the Illumina sequencing there is no significant correlation between coverage and heterozygosity, despite highly variable genome coverage (Table 1). This suggests that heterozygous genotypes are occurring consistently in Illumina sequencing due to consistent and repeatable causes such as misalignment. Furthermore, in a visual inspection of SNPs which are heterozygous in Illumina but homozygous in minION, multiple haplotypes can be observed (Fig. S1), which have likely arisen from sequencing reads originating from duplicated regions within the canola genome but aligning to the same region in the reference genome. In addition, SNPs found within a c. 7525 bp window (the average minION read length in this study) of 55 SNPs which are heterozygous in Illumina but homozygous in minION were manually inspected. It was observed that the majority of the surrounding SNPs are concordant between Illumina and minION (data not shown). Typically, in the Illumina data, a small number of heterozygous SNPs cluster together and are surrounded by regions with fully homozygous SNPs, as genotyped by both technologies. Demonstrating that the heterozygosity in Illumina is generated by local misalignment while the minION reads benefit from diversity in the surrounding regions and so is more likely to correctly position reads. However, within these regions, some genotypes in minION were heterozygous, but the average percentage was below 10%, the current sequencing error rate, suggesting that the minION homozygous genotypes are correct.

**Table 1:** Coverage of the Darmor-*bzh* genome generated in each sample across all sequencing runs and their associated total percentage of heterozygous genotypes.

	Illumina		Minion									
			All runs		Run 1		Run 2		Run 3		Run 4	
	Coverage	% hets	Coverage	% hets	Coverage	% hets	Coverage	% hets	Coverage	% hets	Coverage	% hets
DH-1	7.6	3.7	4.2	4.0	0.9	2.6	0.5	2.0	1.5	2.6	1.4	2.5
DH-2	9.1	4.6	7.4	4.0	0.9	2.8	0.4	2.1	3.0	2.5	3.0	2.5
DH-3	4.9	4.8	2.8	4.4	0.9	2.7	0.6	2.0	0.7	3.1	0.6	3.0
DH-4	8.4	6.1	3.9	4.1	1.0	2.5	0.6	1.8	1.2	2.9	1.1	2.8
DH-5	4.4	5.2	1.7	5.1	1.0	3.0	0.5	2.3	0.1	3.7	0.1	3.6
DH-6	6.3	3.6	2.4	4.0	1.1	2.4	0.5	2.1	0.4	3.2	0.4	3.0
DH-7	12.0	4.8	4.9	4.1	0.9	2.7	0.6	2.1	1.8	2.7	1.7	2.7
DH-8	14.4	4.3	4.9	3.9	1.1	2.5	0.6	1.9	1.6	2.7	1.6	2.6
DH-9	9.5	4.1	1.7	4.2	0.9	2.5	0.5	1.9	0.2	3.5	0.1	2.9
AVERAGE		**4.6**		**4.2**		**2.6**		**2.0**		**3.0**		**2.8**
Correlation with coverage		−0.09		−0.56		−0.15		−0.47		−0.89***		−0.75**

***Signif at 0.01.

**Signif at 0.02.

### Accuracy of genotypes at known SNP positions

**Figure 2:**
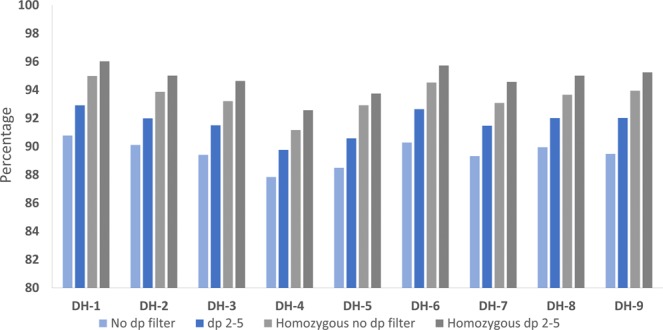
Percentage of accurate genotype calls for each DH sample based on the sequencing data from all 4 minION sequencing runs. Four different filtering treatments were applied: no filtering was performed (no dp filter), a minimum read depth of 2 and a maximum read depth of 5 (dp 2–5), removal of any heterozygous genotype calls in the minION sequences and without depth filtering (homozygous no dp filter) and removal of any heterozygous genotype calls in the minION sequences and a minimum read depth of 2 and a maximum read depth of 5 (homozygous dp 2–5).

**Figure 3:**
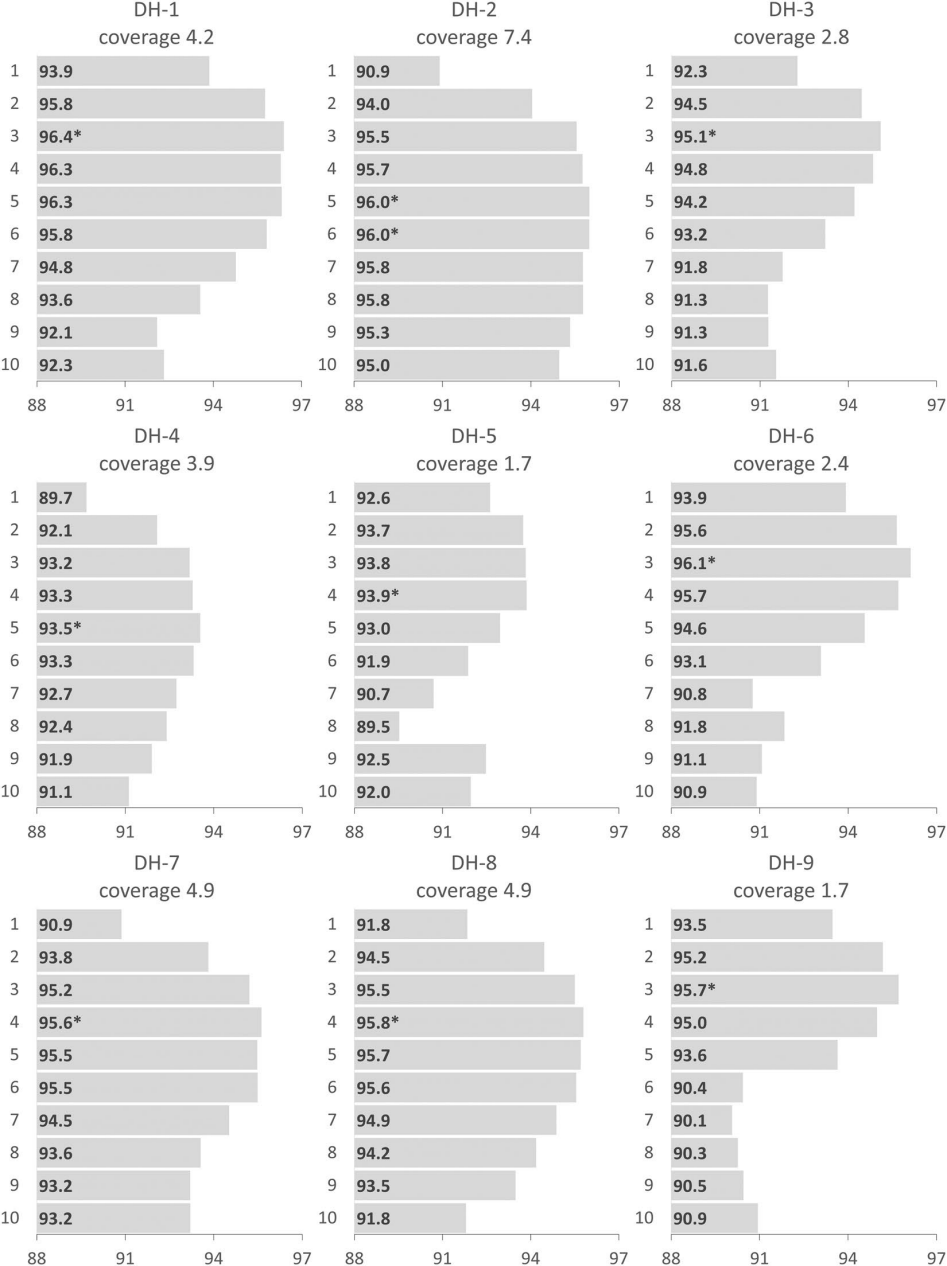
Percentage of accurate genotype calls based on the number of supporting reads. The optimal number of supporting reads is marked with an asterisk (*).

The overall accuracy of minION genotypes was improved in two ways. Firstly, removing all heterozygous genotypes increased accuracy by 2.8–4.5% (Fig. 2). Secondly, filtering for both a minimum and maximum read depth increased accuracy by a further 0.8–2.5%, for a maximum accuracy of 96.0% in DH-1 (Fig. 2). The optimal number of supporting reads is significantly correlated to realised genome coverage (p < 0.05: Fig. 3), such that a read depth filtering range should be determined on a case-by-case basis for maximum benefit. This suggests that the genome is more evenly represented by minION sequencing compared to Illumina sequencing. Employing a tailored depth filtering approach is beneficial for minION genotypes, but the overall level of genome coverage does not correlate to overall accuracy (Fig. S2). There was no benefit to excluding shorter reads or trimming the ends of the reads (Table 2).

**Table 2:** Effect of minimum read length filtering and trimming the ends of minION reads on heterozygosity and accuracy across the all DH samples, filtered for between 2 and 5 supporting reads.

DP 2–5 genotype calls	None	>500 bp reads	>1 kbp reads	>4 kbp reads	100 bp ends trimmed
Average nr of comparable SNPs per individual	779,184	776,426	770,463	708,370	759,754
Discrepant with Illumina %	8.3	8.4	8.4	8.4	8.4
Concordant with Illumina %	91.7	91.6	91.6	91.6	91.6
Heterozygous in minION %	4.2	4.2	4.2	4.2	4.2
**Homozygous genotype calls**					
Discrepant with Illumina %	5.3	5.3	5.3	5.4	5.3
Concordant with Illumina %	94.7	94.7	94.7	94.6	94.7

Examining SNP loci which have been confirmed in multiple populations or sequencing platforms can increase the confidence of SNP calling. In the absence of a SNP database or genotypes from multiple studies and genotyping methods, a subset of c. 900 K markers common between the SNP list used in this study and a set of SNPs identified across diverse *B. napus* (see materials and methods), were examined. The percentage of accurate genotype calls remains similar between the full c. 4 million SNP set used in this study and the 900 K subset (Table S3).

### Accuracy on a per SNP basis

**Figure 4:**
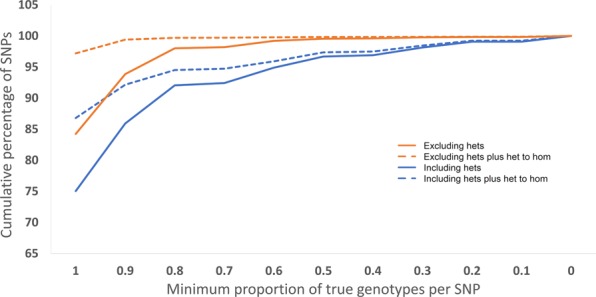
Cumulative percentage frequency of SNPs for the minimum proportion of true genotypes per SNP. The blue lines include heterozygous genotypes calls from the minION data, the orange lines include only homozygous genotype calls. The solid lines represent only genotype calls which are congruent between Illumina and minION, the dashed lines represent all genotype calls which are congruent between the Illumina and minION as well as genotypes which are heterozygous in Illumina but homozygous in minION.

To determine whether errors are more likely to occur in certain SNPs or are randomly spread, accuracy on per SNP basis rather than per individual basis, was also examined. All SNPs were filtered for a read depth between 2 and 5, had to be genotyped in at least 7 of the 9 samples and had to have a non-missing genotype in the corresponding Illumina data. The cumulative percentage frequency of accurate SNPs was calculated for the minimum proportion of individuals with a correct genotype in that SNP (Fig. 4). Examining SNPs when heterozygous minION genotypes are retained (13,708 SNPs), 75% of SNPs are accurately genotyped in all captured individuals, 92% of SNPs are accurate in at least 0.8 of individuals and, 95% of SNPs are accurately genotyped in at least 0.5 of individuals. This can be improved by discarding any heterozygous minION genotype call (12,068 SNPs), where 84.2% of SNPs are accurate in all captured individuals, 93.9% of SNPs are accurate in at least 0.9 of individuals, 98% are accurate in at least 0.8 of individuals. However, some inconsistency is expected between Illumina and minION sequencing due to improved alignment of long reads. As such, genotypes which are heterozygous in Illumina but homozygous in minION are likely to be correctly called from the LRS data, and if these are counted as correctly genotyped, up to 97.2% of SNPs are correct in 100% of individuals (Fig. 4).

### Characterisation of errors

The genotypes which are discrepant between the Illumina and minION sequences were examined to determine the reasons behind this difference. Although discrepant genotypes have mostly been referred to in this study as incorrect in the minION sequencing so far, they are not necessarily incorrect genotypes. Of all the genotype calls in the DH samples, filtered for dp 2–5 and including heterozygotes, 8.3% were discrepant between Illumina and minION sequences. Of these discrepant genotypes, the most common situation was for the genotype to be heterozygous in Illumina but homozygous in minION (43.0%). In this situation, the minION genotypes are more likely to be correct, as it is expected that these samples are fully homozygous. The second most common situation was where the genotype is homozygous in Illumina but heterozygous in minION (38.9%) and may be caused by sequencing error in the minION sequencing. Finally, 18.1% were homozygous in Illumina but the alternative homozygous class in minION. This last situation is more difficult to classify and may be due to the introduction of errors in minION sequencing, however either technology could be correct in this instance. However, it is unlikely that heterozygous minION genotypes will be retained in DHs, and as such, if all heterozygous genotypes are excluded, an examination of discrepant genotype calls (5.3%) found that 70.3% are heterozygous in Illumina but homozygous in minION, and the remaining 29.7% were homozygous in Illumina but the other homozygous class in minION. As these are DHs, the genotypes which are heterozygous in Illumina but homozygous in minION are assumed to be correct in the latter, increasing the total percentage of correct genotypes across all samples from 94.7% to 98.4% (homozygous only, dp 2–5).

Conversely, examining all the Illumina genotypes which are homozygous, 95% are concordant in the minION sequences (Table 3). The majority of false calls are caused by a heterozygous genotype in minION (68.2%). Of the Illumina heterozygous genotype calls, the majority are homozygous in the minION sequences (84.7%) suggesting an improvement due to correct alignment of reads. However, 15.3% of genotypes which are heterozygous in Illumina are also heterozygous in the minION sequences. Overall this represents a very small percentage of all genotypes (0.6%) and this residual heterozygosity may be due to error in the minION sequences, collapsed regions of the reference genome or genuine heterozygosity.

**Table 3:** Analysis of genotype calls which are not consistent between the Illumina and minION sequences, based on whether the genotype call is homozygous or heterozygous in Illumina.

Illumina Genotype	minION Genotype	Percentage
Homozygous	Same genotype as Illumina	95.0
	Discrepant	5.0
	Discrepant and homozygous	*31.8*
	Discrepant and heterozygous	*68.2*
Heterozygous	Same genotype as Illumina	15.2
	Discrepant (i.e. homozygous)	84.7

### Nucleotide bias

**Figure 5:**
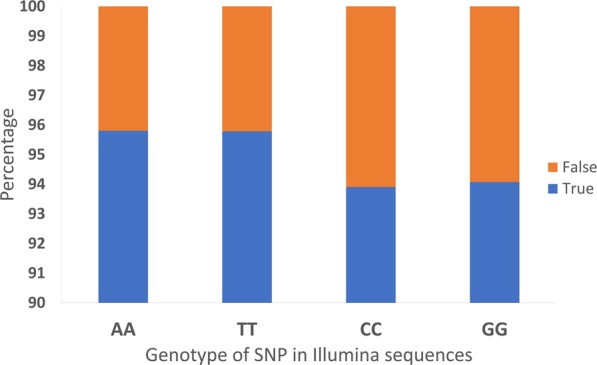
Nucleotide bias in minION sequencing. The percentage of discrepant and correct genotype calls in minION sequencing for each homozygous genotype class captured in the Illumina sequencing.

As previous studies have found a nucleotide bias in minION sequencing, all genotypes which were homozygous in the Illumina sequences were examined based on nucleotide type, and the proportion of discrepant versus correct genotypes determined in the minION sequencing (Fig. 5). SNP genotypes which are CC or GG based on Illumina sequencing contain a higher percentage of discrepancies in the minION sequencing, but this is not statistically significant. Restricting the pre-defined SNP list to only include genotypes which are only potentially an A or T nucleotide does not significantly alter accuracy, with an average accuracy across all samples of 94.7% from the full list and 94.9% in the restricted list, both filtered for homozygous SNPs at depth 2 to 5. Therefore, although the nucleotide bias of minION sequencing can be observed, the effect on overall genotyping accuracy is minimal and unlikely to be an issue.

### Approaches to improve the accuracy of genotype calls in heterozygous material

Although the simplest approach is to discard any heterozygous genotype calls, this is only feasible in fully homozygous samples. As plant breeding often uses heterozygotes, approaches to improve the accuracy of heterozygous genotype calls was examined. Firstly, VCF files contain PL values, describing the phred-scaled likelihoods of the possible genotypes, with a value of 0 indicating the most likely genotype. There are scenarios where a SNP has been called heterozygous, but the most likely genotype is one of the homozygous genotypic classes according to the PL values, which is calculated based on numerous factors. Converting heterozygous genotypes to the homozygous genotype with the best PL value was tested. A total of 272,940 genotypes were heterozygous in minION across all SNP loci and individuals (dp 2–5), and if minION heterozygous calls are converted to the homozygous genotype class with the lowest PL value when the heterozygous PL value is not 0 (the most likely genotype), 41.3% of minION heterozygotes are converted to a homozygous class. Of these PL adjusted genotypes, 67.8% are concordant, 25% discrepant due to heterozygosity in Illumina and 7.2% are discrepant as the Illumina genotype is the alternative homozygous class.

Secondly, the allelic depth of the reference and alternative allele can be used to adjust heterozygous genotypes. As such an approach is likely to require greater sequencing depth, it was tested at regions with more supporting reads, dp 4–6, as well as dp 2–5. Filtering for regions with more supporting reads did not improve accuracy of allelic depth-based adjustment (Table S4), perhaps because the overall accuracy of genotype calls is highly correlated with the expected sequencing depth (Fig. 3), with most samples in this study optimal at between 2 and 5 supporting reads. As compared to a PL value-based approach, at a read depth range between 2 and 5, 71.1% of heterozygotes were converted, achieving marginally lower accuracy, with a higher proportion of adjusted genotypes which are discordant due to the other homozygous genotype class being present in the Illumina sequencing (Table S4).

Lastly, the effect of altering the minimum base quality score required by Samtools mpileup for inclusion in genotyping calling was examined. The default score of 13, as has been used throughout this study, was compared to a less stringent minimum base score of 1, and a more stringent minimum of 20. Applying harsher minimum base quality score does marginally improve the overall concordance of genotype calls between Illumina and minION sequencing (Table S5). However, there is a significant reduction in the average number of loci, such that the marginal increase in accuracy is outweighed by the loss of markers.

## Discussion

Unpolished ONT minION LRS has the potential to be used as a SNP genotyping platform when using a list of known SNP loci, rather than *de novo* SNP discovery, but will require substantial advances in sequencing accuracy or bioinformatic handling before it is suitable for routine SNP genotyping outside of homozygous germplasm. Although the majority of *B. napus* cultivars are DHs or highly homozygous due to their breeding history, filtering on excess heterozygosity will not always be feasible, as some genuine heterozygosity is likely present, including recent alleles which have not yet become fixed, and it may be necessary to genotype heterozygous populations. Ultimately, the accuracy of association studies and genomic prediction is increased when all present variation is captured, including heterozygotes^17–19^. At current error rates and using the simple genotype calling techniques employed in this study, it is necessary to remove all heterozygous genotype calls to achieve sufficient accuracy for use in downstream applications. Unless employed in completely homozygous genomes, methods to distinguish between true heterozygotes and false positives need to be developed, preventing the implementation of this method in species and samples expected to display heterozygous genotypes.

Although heterozygous samples will be bioinformatically more difficult, structured heterozygous populations will be easier to work with and are likely to benefit from the techniques currently applied in skim WGR of bi-parental populations, particularly in rice^37,38^. The extremely low sequencing depth requires the use of advanced bioinformatic techniques including a sliding-window approach to correctly assign genotypes based on either known or inferred parental genotypes. Another potential technique would be to use a haplotype-based approach, assigning a parental haplotype to each sample rather than genotyping individual SNPs and reducing the effect of individual sequencing errors as long as selected haplotypic regions are sufficiently divergent.

MinION LRS avoids biases which affect other SRS systems including PCR bias, GC-content and mappability^30^, and for these reasons results in a more even representation of the genome. It appears that the kurtosis of the normal distribution of genome coverage is reduced in minION compared to Illumina sequencing, such that the significant variation in realised sequencing coverage across the genome which is observed in other SRS systems^39^ is not present to the same extent. The effect of this was clearly seen on the accuracy of genotype calls at different numbers of supporting reads, with a perceptible decrease in accuracy at greater than expected coverage levels. This highlights the importance of interpreting sequencing data with the effect of sequencing platform in mind and in the case of minION LRS, results in a benefit to filtering depth around realised coverage, with the optimal number of supporting reads correlating with coverage.

Another advantage of LRS over SRS is the potential for the improvement of sequencing read alignment in highly duplicated genomes. Although there is not a significant reduction in the number of heterozygous genotype calls in the minION LRS in comparison to Illumina SRS, the relatively high negative correlation between genome coverage and heterozygosity in minION, as opposed to Illumina, is indicative of improved alignment accuracy. Essentially, the issue of identifying erroneous SNPs caused by misalignment is removed before the SNP calling step is performed. This suggests that the heterozygous genotypes observed in the minION LRS are a result of sequencing error, as heterozygosity is reduced with additional sequencing depth. Although nanopore sequencing results in some systemic errors, it results in errors which are more randomly spread throughout the genome than those caused by misalignment. Random errors are less likely to impact downstream applications compared to consistent errors^40,41^. Conversely, increasing sequencing coverage did not reduce heterozygosity in the Illumina SRS and is consistent with the high accuracy but short read length of Illumina sequencing resulting in misalignment. In addition, common quality filtering applied to SRS aimed at minimising this issue removes a large proportion of reads which cannot be uniquely aligned to the reference genome, as was observed in this study a well as others^11,42,43^. As the nanopore long reads cannot be quality filtered in the same way due to the high overall error rate, more of the sequencing data is utilised and therefore, potentially less sequencing data can generate the same outcome compared to traditional SRS systems. An investigation into the authenticity of SNPs identified by ddRAD found 41% of fragments did not yield expected results due to the presence of homologous sequences^44^, which is likely the primary cause of genotyping errors when using SRS in duplicated genomes. Both short reads and probes have been repeatedly shown to struggle with sequence similarity, resulting in frequent misalignment in duplicated genomes including canola^5–8,11,15,16,44,45^ but many other crop species are also highly duplicated, including soybean^46,47^, maize^12^, peanut^48^ and cotton^49–51^. Duplication is prevalent across all plant species, with plant genomes containing an average of 64.5% paralogous genes^52^ and therefore it is likely that the development of LRS based genotyping protocols will be of benefit across not only crop species but all plants.

Despite this improvement in read alignment, numerous heterozygous genotypes are observed in the minION sequencing, with a small number of genotypes found to be heterozygous in both sequencing systems. Although heterozygous genotype calls in DHs can be indicative of misalignment, there are other possible causes. Firstly, sequencing error can cause heterozygous genotypes, particularly in low coverage samples lacking sufficient reads to drown out noise^35,53^, and is especially relevant for minION, which has a higher error rate than current SRS technologies^30,31^. Secondly, sequence collapse, incomplete or missing regions of the reference genome resulting in the absence of one or more duplicated regions may lead to heterozygous genotype calls. For instance, if there are two highly similar regions in the genome, but only one is present in the reference sequence, all reads arising from both of these homoeologues will align uniquely to a single region of the reference and may lead to false-positive SNPs if there is differentiation between the two regions. Additionally, gene transfer from organelles to nuclear DNA is common^54^ such that organelle DNA will align to these regions and may cause heterozygotes. Such misalignment can only be removed by incorporating the organelle sequences into the reference genome. Lastly, these genotypes may be genuinely heterozygous, as the process of producing DHs is not always completely effective^55^. Currently, the most practical method is to remove any heterozygous genotypes, but this approach will not be suitable in genomes expected to contain genuine heterozygosity. Additionally, c. 1.5% of all genotype calls were one homozygous genotype class in the Illumina SRS and the alternate homozygous genotype class in the minION LRS. Genotypes of this sort cannot be confidently classified as correct or incorrect, nor can they be identified in data sets based only on a single sequencing platform. Thankfully this represents only a small percentage of genotype calls but will inevitably be included in any SNP data sets generated from minION sequencing.

As minION LRS results in improved alignment in duplicated genomes, complete concordance between Illumina and minION sequencing is not expected. Instead, by calculating the accuracy of minION sequencing as the percentage of homozygous genotype calls which are the same in both systems plus the genotypes which are heterozygous in Illumina SRS but homozygous in minION LRS, accuracy as high as 98.4% can be achieved in nanopore LRS of DHs. For context, genotypes compared between more deeply sequenced (10x genome coverage) Illumina SRS samples and those same samples with skim sequencing coverage can contain between 1.2% discrepant genotypes at 5x coverage and 7.7% discrepant genotypes at 0.25x coverage^35^. Additionally, choice of SNP calling software has a substantial effect on genotypes^56^, with concordance ranging anywhere from c. 92% to over 99%^57,58^.

A number of techniques commonly applied to improve minION LRS for other applications, such as *de novo* assembly, were examined but did not affect genotyping accuracy. It has become common practise to improve nanopore based *de novo* assemblies by polishing the sequences with more accurate SRS as it greatly improves sequence accuracy and consequently genome completeness^59–62^. However, the amount of Illumina SRS required for polishing will also likely be sufficient for SNP genotyping in most species^35^, such that any nanopore LRS based genotyping strategy must be sufficiently accurate without polishing. In addition, polishing involves the alignment of SRS to the LRS and will have similar issues with misalignment between homoeologous regions as observed when aligning SRS to a reference genome and may introduce errors which will appear to be SNPs. Recent studies have found that nanopore sequencing errors are not completely random, with a substitution bias of G and C nucleotides, and the deletion bias for A and T nucleotides^30,63^. The effect of this on overall genotyping accuracy was negligible with minimal improvement when using a restricted list of A or T only SNPs. Nor did trimming the more error prone ends of reads^31^ have any effect since, realistically, removing a total of 200 bp from reads averaging 7,525 bp in length (Q >= 7) equates to approximately 2.7% of sequencing data, such that there is minimal likelihood of pre-define SNPs falling within the flanking regions of reads. Additionally, nanopore LRS based *de novo* assemblies have benefitted from targeting longer read fragments^29^ and excluding reads below a minimum length threshold, resulting in less fragmented assemblies despite incorporating less sequencing data overall^61^, but this approach did not improve overall genotyping accuracy. Even small fragments (c. 500 bp) sequenced on the minION are longer than the 150–250 bp produced by Illumina sequencing and provide sufficiently long reads for improved alignment to the reference genome.

As there is no benefit in excluding shorter fragments, more sequence reads can be utilised for genotyping and efforts to increase fragment length at the DNA extraction and size selection steps can largely be omitted. Nonetheless, input DNA quality should be considered a priority as it has been consistently found to have a significant effect on sequencing yield^29,64^. This study found the QIAGEN DNeasy 96 Plant Kit to produce sufficiently high quality DNA, such that the ability to use cost effective, high-throughput DNA extraction methods, coupled with relatively quick library preparation (less than one day) suggests ONT LRS has the potential to become a routine genotyping method. However, ONT library preparation protocols currently have a number of limitations that would prevent the adoption of this technology for high-throughput genotyping. Currently, there are only 24 ligation barcodes available for minION and promethION, and while there are 96 PCR-based barcodes available for minION sequencing, the use of PCR will introduce classic PCR biases which also effect Illumina sequencing. Similarly, sequencing outputs are increasing steadily but do not yet match the same price structure as Illumina.

Bioinformatic tools for ONT sequencing face similar issues, as some software does exist for variant genotyping, but is often originating from SRS and has not been optimised for LRS. However, specific software does exist including marginCaller^65^, which was found to have a high detection rate of true SNPs but has a substantial false positive rate which is affected by GC error bias issues^30^, and currently does not allow genotyping of known variant sites or homozygous reference genotypes. The consensus-based approach of Nanopolish^66^ has been found to result in highly accurate genotyping of pre-defined SNPs. For instance, applying Nanopolish for variant genotyping of c. 30x coverage of the human genome allowed 99.16% of genotypes to be called correctly, including many homozygous reference sites, which translated to 94.83% accuracy of variant genotypes^29^. In order to minimise the effect of noise, this study required the log-likelihood ratio of a variant call to be at least 30, or it was considered to be homozygous reference. These methods may require substantial sequencing depth, and in the case of Nanopolish, requires the fast5 sequences which are substantially larger than fastq/a formats. The development of genotyping algorithms with the behaviour of ONT sequencing error and approaches recalling heterozygous genotypes based on allelic proportions, which was found to be moderately accurate in this study, could be of benefit but may require greater sequencing coverage to ensure recovery of true allelic proportions. Adjustment of heterozygous genotypes based on genotype likelihoods defined in the VCF PL field was also moderately accurate. However, less than half of heterozygous calls were converted, while in comparison, adjusting based on allelic depth resulted in similar accuracy but converted just over 70% of heterozygous calls. Nonetheless, PL values are calculated based on a number of factors, such as base quality, allelic depth and population data, making this approach reasonable and computationally simple for minION users, and may be improved by the use of larger sample sets. Additionally, filtering minION reads on a higher minimum Q score cut-off than the default of 7 used in this study may have some benefit. At the genotype calling stage, solely applying a higher minimum base quality during genotype calling did marginally improve the overall accuracy but was outweighed by the significant reduction in marker numbers. With the steady increase in sequencing accuracy likely to continue as sequencing chemistry and pore technology improves, the development of easy to implement bioinformatic strategies for genotyping of ONT LRS is probable.

## Conclusion

Even with the simple bioinformatic pipeline used in this study, overall genotype accuracy as high as 98.4% is the maximum accuracy achieved, as stated in the results. 97.2% is the maximum percentage of SNPs which are correct in all individuals under optimal filtering conditions and assumptions was achieved in a set of 9 DH canola samples. More advanced approaches can realistically be expected to lead to reliable genotyping of even more complex germplasm in the future. Currently, this technology could reasonably be used to genotype known SNP loci in DHs and the improvement of existing SNP lists in species with highly duplicated genomes. The availability of some genomic resources, specifically in the form a reference genome and a pre-defined list of SNPs is still required. The expected cost-structure and throughput of ONT sequencing systems in the near future is promising not only for canola but many crop and plant species of interest, due improvement in misalignment between duplicated regions and more even representation of the genome.

## Materials and Methods

### Plant material and DNA extraction

Genomic DNA was extracted from leaf tissue of 9 Australian spring type *B. napus* DHs using the DNeasy 96 Plant Kit (QIAGEN, Hilden, Germany), according to the manufacturer’s instructions. Multiple elutions per sample were pooled and concentrated using a 1.8x ratio of Agencourt AMPure XP beads (Beckman Coulter, Pasadena, CA, United States). Resulting DNA was not size selected.

### Library preparation and sequencing

Libraries were prepared for minION sequencing according to the manufacturers protocol, using the Native Barcoding expansion pack (EXP-NBD103) and the 1D Sequencing kit, with either the SQK-LSK108 or SQK-LSK109 chemistry. No additional shearing was performed, but the DNA repair step was performed according to the protocol, starting with 1.5 µg of DNA per sample. A total of 4 flowcells (FLO-MIN106) were each run for the full 48 hours, generating c. 7 Gbp (SQK-LSK108), c. 4 Gbp (SQK-LSK108), c. 10 Gbp (SQK-LSK109) and c. 9 Gbp (SQK-LSK109) of sequencing respectively.

Each of the 9 DH samples were also whole genome sequenced to between 4.4–14.4x coverage on an Illumina Hiseq3000 using a previously described library preparation method for whole genome sequencing of DHs^35^.

### Bioinformatics

Basecalling was performed using Albacore (v2.3.1) for all minION sequencing data, applying the default minimum Q score of 7. Adapters were trimmed using Porechop (v0.2.3) and a range of minimum read lengths applied using Filtlong (v0.2.0) to determine whether minimum read length has any effect on overall genotyping accuracy. End trimming was performed using cutadapt^67^ to determine whether trimming the ends of reads had any effect on accuracy.

The resulting fastq files were aligned to the Darmor-*bzh* whole genome reference^1^ using minimap2^68^ and converted to BAM files using SAMtools view^69^. SNPs were called using SAMtools mpileup (v1.3.1) supplied with a list of c. 4 million pre-validated SNPs^16^ and converted to a VCF using BCFtools call^69^ and VCFtools^70^, ignoring indels and removing triallelic SNPs but otherwise retaining all genotypes whether variant or not. Resulting VCF files were converted to genotype and depth matrices, and the accuracy of genotype calls in the long reads sequences was evaluated in R^71^. The minION-based genotype calls were compared to the genotype calls of the same samples sequenced using Illumina SRS and filtered to a minimum read depth of 2 unless otherwise stated. Not all SNPs could be compared due to missing data in either the LRS or SRS sequences, and so were excluded from this analysis. As filtering for both a minimum and maximum read depth was found to be beneficial for minION sequencing, instances where all samples were required to undergo the same treatment for consistency, a read depth range between 2 and 5 supporting reads was applied due to the balance between overall accuracy and number of available data points. Alignments of sequencing reads to the reference genome were visualised using either Tablet^72^ or IGV^73^. Adjustment of heterozygous genotypes based on PL value and allelic depth were performed in R, by extracting the PL and AD fields from the VCF file.

To address the possibility that the SNP positions used in this study may contain some loci which are not genuine, c. 900 K SNPs which were common between the SNP list used in this study^16^ and set of SNP loci which were also identified across the diversity of canola^74^ were examined. There is higher confidence that these SNPs are true variants in canola as they’ve been independently verified in two studies, across a range of germplasm types.

## Supplementary information

Supplementary Materials

## Data Availability

The sequences generated during the current study are available from links in the NCBI BioProject database under BioProject accession number PRJNA517536.
