# Supplementary material for: Assessment of low-coverage nanopore long read sequencing for SNP genotyping in doubled haploid canola (Brassica napus L.)
Source: Sci Rep. 2019 Jun 18;9:8688. doi: 10.1038/s41598-019-45131-0 (PMC6582154; doi:10.1038/s41598-019-45131-0)
Supplement: Supplementary file 1 — Supplementary Materials [file 41598_2019_45131_MOESM1_ESM.docx]

Supplementary Material

Assessment of low-coverage nanopore long read sequencing for SNP genotyping in doubled haploid canola (*Brassica napus* L.)

M.M. Malmberg, G.C. Spangenberg, H.D. Daetwyler and N.O.I. Cogan

|  |  |  | **All reads** |  |  |  | **Q>=7** |  |  |  |
| --- | --- | --- | --- | --- | --- | --- | --- | --- | --- | --- |
| **Run** | **Samples** | **Chemistry** | **Reads** | **Gb** | **Mean length (bp)** | **Mean Q** | **Reads** | **Gb** | **Mean length (bp)** | **Mean Q** |
| **1** | **9** | 108 | 822,968 | 7.0 | 8446.3 | 9.3 | 741,711 | 6.5 | 8728.3 | 9.7 |
| **2** | **9** | 108 | 560,249 | 4.1 | 7356.5 | 9.2 | 496,657 | 3.8 | 7614.5 | 9.7 |
| **3** | **9** | 109 | 1,388,167 | 9.0 | 6480.6 | 9.3 | 1,226,206 | 8.3 | 6758.2 | 9.9 |
| **4** | **9** | 109 | 1,231,813 | 8.2 | 6694.5 | 9.5 | 1,097,112 | 7.7 | 7000.3 | 10 |

**Table S1:** Quality metrics for each of the barcoded minION sequencing runs (runs 1-4).

|  | **% of reads mapped** | |
| --- | --- | --- |
|  | **Illumina (q30)** | **minION** |
| **DH-1** | 59.1 | 98.7 |
| **DH-2** | 54.7 | 98.8 |
| **DH-3** | 51.1 | 98.3 |
| **DH-4** | 57.9 | 97.5 |
| **DH-5** | 64.2 | 98.1 |
| **DH-6** | 54.3 | 98.2 |
| **DH-7** | 57.9 | 98.8 |
| **DH-8** | 66.0 | 98.7 |
| **DH-9** | 54.3 | 98.6 |

**Table S2**: Percentage of sequencing reads which aligned to the Darmor-*bzh* reference genome. The Illumina SRS have been filtered for a minimum q score of 30.

Figure S1


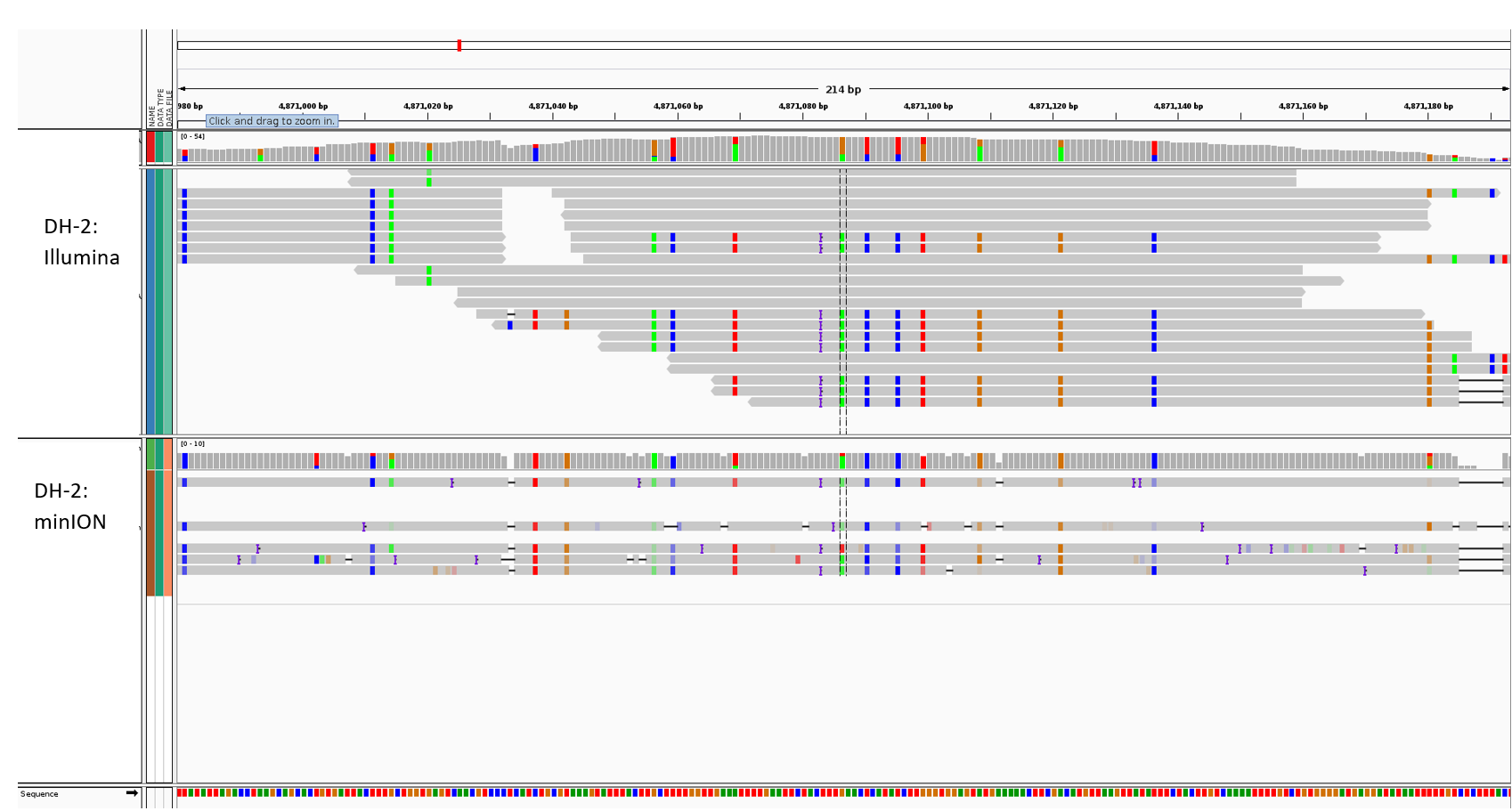


**Figure S1:** A view in IGV of SNP chrA05_POS_4871086 (indicated by dotted lines) in sample DH-2 showing alignment of multiple haplotypes to the reference genome, likely due to reads arising from multiple regions of the genome but aligning to the same region in the reference genome.

Figure S2


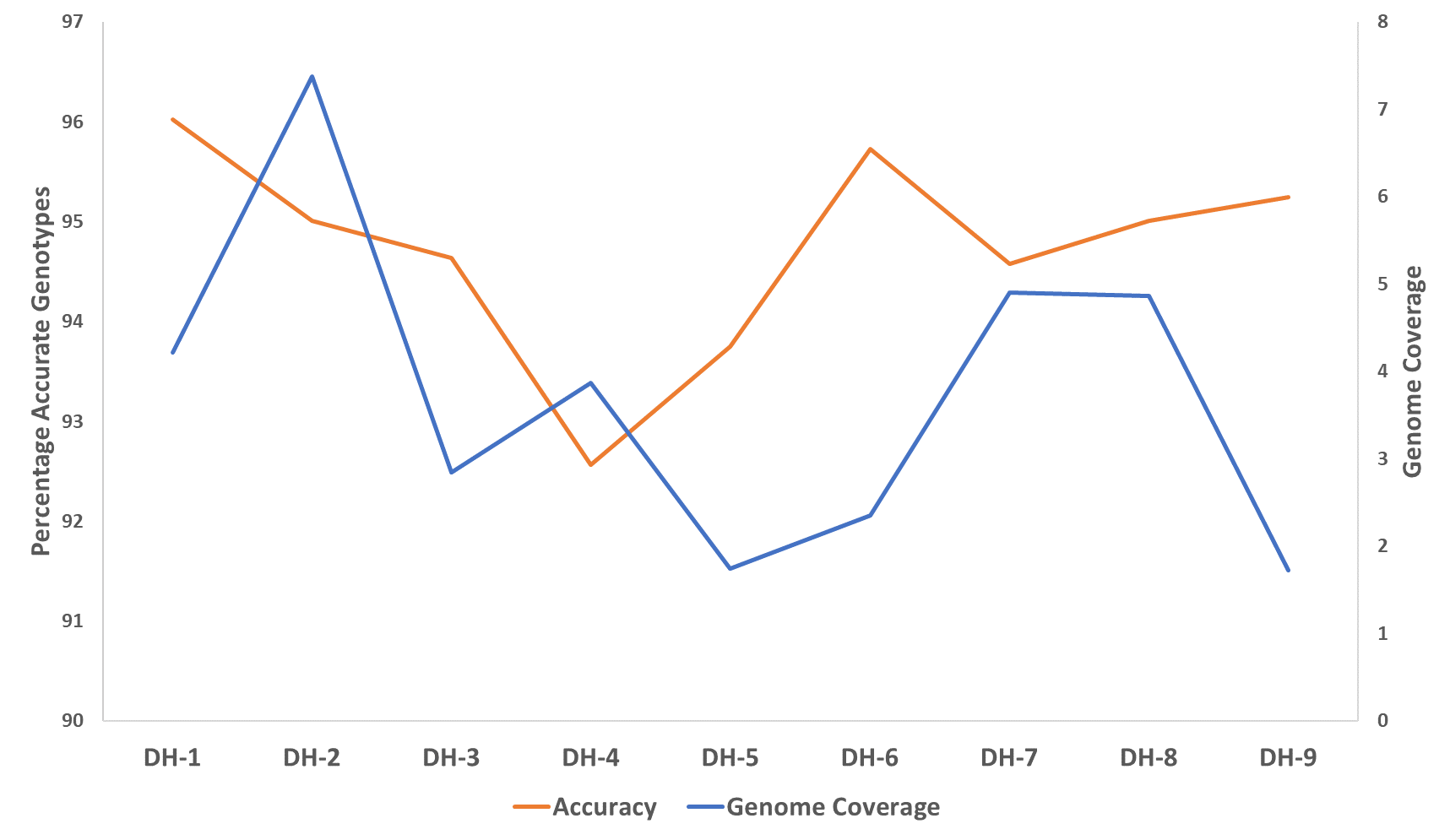


**Figure S2:** Plot of the percentage of accurate genotype calls (orange line) in each DH sample filtered to keep homozygous genotype calls only and with a read depth between 2-5 reads, against total genome coverage (blue line).
